# Megadrought and Megadeath in 16th Century Mexico

**DOI:** 10.3201/eid0804.010175

**Published:** 2002-04

**Authors:** Rodolfo Acuna-Soto, David W. Stahle, Malcolm K. Cleaveland, Matthew D. Therrell

**Affiliations:** * Universidad Nacional Autonoma de Mexico, Ciudad Universitaria, Mexico; † University of Arkansas, Fayetteville, Arkansas, USA

## Abstract

The native population collapse in 16th century Mexico was a demographic catastrophe with one of the highest death rates in history. Recently developed tree-ring evidence has allowed the levels of precipitation to be reconstructed for north central Mexico, adding to the growing body of epidemiologic evidence and indicating that the 1545 and 1576 epidemics of cocoliztli (Nahuatl for "pest”) were indigenous hemorrhagic fevers transmitted by rodent hosts and aggravated by extreme drought conditions.

The native people of Mexico experienced an epidemic disease in the wake of European conquest (Figure 1), beginning with the smallpox epidemic of 1519 to 1520 when 5 million to 8 million people perished. The catastrophic epidemics that began in 1545 and 1576 subsequently killed an additional 7 million to 17 million people in the highlands of Mexico (*1*–*3*). Recent epidemiologic research suggests that the events in 1545 and 1576, associated with a high death rate and referred to as cocoliztli (Nahuatl for "pest"), may have been due to indigenous hemorrhagic fevers (*4*,*5*). Tree-ring evidence, allowing reconstructions of the levels precipitation, indicate that the worst drought to afflict North America in the past 500 years also occurred in the mid-16th century, when severe drought extended at times from Mexico to the boreal forest and from the Pacific to Atlantic coasts (*6*). These droughts appear to have interacted with ecologic and sociologic conditions, magnifying the human impact of infectious disease in 16th-century Mexico.

**Figure 1:**
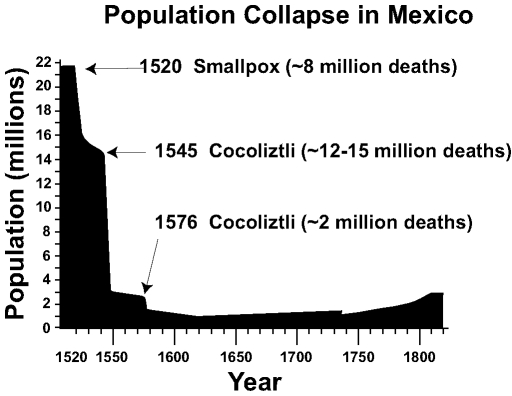
The 16th-century population collapse in Mexico, based on estimates of Cook and Simpson (*1*). The 1545 and 1576 cocoliztli epidemics appear to have been hemorrhagic fevers caused by an indigenous viral agent and aggravated by unusual climatic conditions. The Mexican population did not recover to pre-Hispanic levels until the 20th century.

The epidemic of cocoliztli from1545 to 1548 killed an estimated 5 million to 15 million people, or up to 80% of the native population of Mexico (Figure 1). In absolute and relative terms the 1545 epidemic was one of the worst demographic catastrophes in human history, approaching even the Black Death of bubonic plague, which killed approximately 25 million in western Europe from 1347 to 1351 or about 50% of the regional population.

The cocoliztli epidemic from 1576 to 1578 cocoliztli epidemic killed an additional 2 to 2.5 million people, or about 50% of the remaining native population. Newly introduced European and African diseases such as smallpox, measles, and typhus have long been the suspected cause of the population collapse in both 1545 and 1576 because both epidemics preferentially killed native people. But careful reanalysis of the 1545 and 1576 epidemics now indicates that they were probably hemorrhagic fevers, likely caused by an indigenous virus and carried by a rodent host. These infections appear to have been aggravated by the extreme climatic conditions of the time and by the poor living conditions and harsh treatment of the native people under the encomienda system of New Spain. The Mexican natives in the encomienda system were treated as virtual slaves, were poorly fed and clothed, and were greatly overworked as farm and mine laborers. This harsh treatment appears to have left them particularly vulnerable to epidemic disease.

Cocoliztli was a swift and highly lethal disease. Francisco Hernandez, the Proto-Medico of New Spain, former personal physician of King Phillip II and one of the most qualified physicians of the day, witnessed the symptoms of the 1576 cocoliztli infections. Hernandez described the gruesome cocoliztli symptoms with clinical accuracy (*4*,*5*). The symptoms included high fever, severe headache, vertigo, black tongue, dark urine, dysentery, severe abdominal and thoracic pain, large nodules behind the ears that often invaded the neck and face, acute neurologic disorders, and profuse bleeding from the nose, eyes, and mouth with death frequently occurring in 3 to 4 days. These symptoms are not consistent with known European or African diseases present in Mexico during the 16th century.

The geography of the 16th century cocoliztli epidemics supports the notion that they may have been indigenous fevers carried by rodents or other hosts native to the highlands of Mexico. In 1545 the epidemic affected the northern and central high valleys of Mexico and ended in Chiapas and Guatemala (*4*). In both the 1545 and 1576 epidemics, the infections were largely absent from the warm, low-lying coastal plains on the Gulf of Mexico and Pacific coasts (*4*). This geography of disease is not consistent with the introduction of an Old World virus to Mexico, which should have effected both coastal and highland populations.

Tree-ring evidence, reconstructed rainfall over Durango, Mexico during the 16th century (*6*), adds support to the hypothesis that unusual climatic conditions may have interacted with host-population dynamics and the cocoliztli virus to aggravate the epidemics of 1545 and 1576. The tree-ring data indicate that both epidemics occurred during the 16th century megadrought, the most severe and sustained drought to impact north central Mexico in the past 600 years (Figure 2; [*7*]). The scenario for the climatic, ecologic, and sociologic mediation of the 16th-century cocoliztli epidemics is reminiscent of the rodent population dynamics involved in the outbreak of hantavirus pulmonary syndrome caused by Sin Nombre Virus on the Colorado Plateau in 1993 (*8*,*9*). Cocoliztli was not pulmonary and may not have been a hantavirus but may have been spread by a rodent host. If true, then the prolonged drought before the 16th-century epidemics would have reduced the available water and food resources. The animal hosts would then tend to concentrate around the remnants of the resource base, where heightened aggressiveness would favor a spread of the viral agent among this residual rodent population. Following improved climatic conditions, the rodents may have invaded both farm fields and homes, where people were infected through aspiration of excreta, thereby initiating the cocoliztli epidemic. The native people of Mexico may have been preferentially infected because they worked the agricultural fields and facilities that were presumably infested with infected rodents.

**Figure 2:**
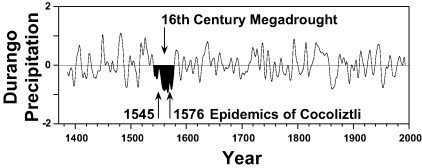
Winter-spring precipitation reconstructed from tree ring data, Durango, Mexico (normalized and smoothed to highlight decennial variability). The tree-ring estimates explain 56% of the variance in precipitation for Durango and are consistent with independent precipitation data. This reconstruction is well correlated with the all-Mexico rainfall index (r = 0.76; p < 0.001) and with precipitation over north central Mexico, where the cocoliztli epidemics appear to have been most severe. Note the unprecedented 16th-century megadrought during both cocoliztli epidemics.

Ten lesser epidemics of cocoliztli began in the years 1559, 1566, 1587, 1592, 1601, 1604, 1606, 1613, 1624, and 1642 (*10*). Nine of them began in years in which the tree-ring reconstructions of precipitation indicate winter-spring (November-March) and early summer (May-June) drought (*8*). But the worst epidemic of cocoliztli ever witnessed, 1545-1548, actually began during a brief wet episode within the era of prolonged drought (Figure 3). This pattern of drought followed by wetness associated with the 1545 epidemic is very similar to the dry-then-wet conditions associated with the hantavirus outbreak in 1993 (Figure 3; [*9*]), when abundant rains after a long drought resulted in a tenfold increase in local deer mouse populations. Wet conditions during the year of epidemic outbreak in both 1545 and 1993 may have led to improved ecologic conditions and may also have resulted in a proliferation of rodents across the landscape and aggravated the cocoliztli epidemic of 1545-1548.

**Figure 3:**
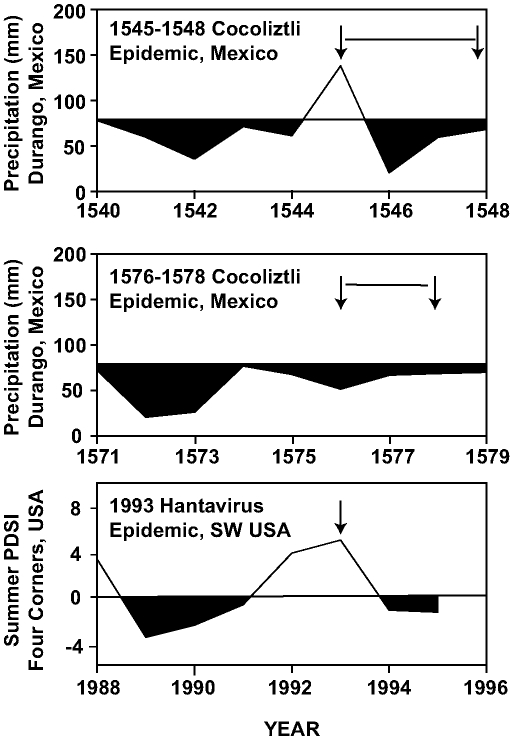
The winter-spring precipitation totals estimated for each year in Durango, 1540–1548 (top), 1571–1579 (middle). Compared with the Palmer drought index, southwestern USA 1988–1995 (bottom). A tenfold increase in deer mice was witnessed in the southwestern USA during the 1993 outbreak, a year of abundant precipitation following a prolonged drought. The similar dry-wet pattern reconstructed for the 1545 epidemic of cocoliztli may have impacted the population dynamics of the suspected rodent host to aggravate the epidemic.

The disease described by Dr. Hernandez in 1576 is difficult to link to any specific etiologic agent or disease known today. Some aspects of cocoliztli epidemiology suggest that a native agent hosted in a rain-sensitive rodent reservoir was responsible for the disease. Many of the symptoms described by Dr. Hernandez occur to a degree in infections by rodent-borne South American arenaviruses, but no arenavirus has been positively identified in Mexico. Hantavirus is a less likely candidate for cocoliztli because epidemics of severe hantavirus hemorrhagic fevers with high death rates are unknown in the New World. The hypothesized viral agent responsible for cocoloztli remains to be identified, but several new arenaviruses and hantaviruses have recently been isolated from the Americas and perhaps more remain to be discovered (*11*). If not extinct, the microorganism that caused cocoliztli may remain hidden in the highlands of Mexico and under favorable climatic conditions could reappear.
